# Bilateral Vein of Trolard Thrombosis Presenting with Seizure and Minimal Deficits: A Rare Case Report

**DOI:** 10.3390/neurolint18080155

**Published:** 2026-08-20

**Authors:** Balaganesh Natarajan, Mahika Khurana, Mariam Gabadadze, Ahmed Abd Elazim, Eman Elmasry Eldamarany Khalifa

**Affiliations:** 1 Department of Neurology, Sanford Medical Center, University of South Dakota Sanford School of Medicine, Sioux Falls, SD 57105, USA; mahika.khurana@usd.edu (M.K.); mariam.gabadadze@usd.edu (M.G.); ahmed.abdelazim@sanfordhealth.org (A.A.E.); 2 Maternity and Children Hospital, Hofuf 36422, Saudi Arabia; elmasry.eman@yahoo.com

**Keywords:** bilateral vein of Trolard thrombosis, isolated cortical vein thrombosis, cerebral venous thrombosis, seizure, cord sign, magnetic resonance venography, subarachnoid hemorrhage

## Abstract

Isolated cortical vein thrombosis is a rare subtype of cerebral venous thrombosis with highly variable clinical and radiologic manifestations that frequently delay diagnosis. Bilateral thrombosis of the veins of Trolard is exceptionally uncommon, with only a few cases reported in the literature. We report a 64-year-old right-handed man who presented after being found unresponsive with suspected seizure. Although his neurological examination was normal at evaluation (NIHSS 0), noncontrast CT demonstrated subtle bilateral cortical vein hyperdensities (cord sign). Subsequent MRI and MR venography confirmed bilateral thrombosis of the veins of Trolard with associated venous congestion and a small sulcal subarachnoid hemorrhage. The patient was treated with therapeutic anticoagulation and levetiracetam. The patient achieved an excellent functional outcome (modified Rankin Scale score of 0). At 3-month follow-up, he remained neurologically intact without recurrent seizures. Follow-up MRI demonstrated improvement of the cortical FLAIR abnormality, and MR venography showed significant interval improvement in the bilateral vein of Trolard thromboses with residual short-segment nonocclusive filling defects, consistent with partial venous recanalization. This case expands the recognized clinical spectrum of the bilateral vein of Trolard thrombosis by demonstrating that extensive bilateral cortical venous involvement may present predominantly with seizure despite a normal neurological examination (NIHSS 0). Early recognition of subtle CT findings, confirmation with dedicated venous imaging, and prompt anticoagulation can result in excellent clinical recovery and favorable radiographic evolution.

## 1. Introduction

Accounting for approximately 0.5–1% of all strokes, cerebral venous thrombosis (CVT) is an uncommon but clinically significant cerebrovascular disorder characterized by variable clinical and radiologic manifestations that frequently delay diagnosis [1,2]. Among its subtypes, isolated cortical vein thrombosis (ICVT) represents only a small proportion of CVT cases and remains one of its least recognized forms because thrombosis is confined to cortical veins rather than the major dural venous sinuses [3,4]. The resulting impairment of superficial cortical venous drainage produces a heterogeneous clinical spectrum that depends on the specific cortical vein involved, the degree of collateral venous compensation, and the severity of venous congestion, ranging from isolated seizures to focal neurological deficits, venous infarction, or intracranial hemorrhage [2,3,4]. Patients therefore often present without the classic radiographic or clinical features associated with dural sinus thrombosis, contributing to under-recognition and misdiagnosis, particularly when neurological deficits are minimal [1]. Common etiologies of CVT include inherited or acquired thrombophilias, malignancy, infection, inflammatory disorders, pregnancy, oral contraceptive use, and trauma; however, isolated cortical vein thrombosis may occur without an identifiable predisposing factor [1].

Seizure is among the most common presenting features of cortical vein thrombosis and may occur in the absence of focal deficits, complicating early diagnostic efforts [2,4]. Furthermore, early imaging findings can be subtle; noncontrast CT may demonstrate a hyperdense cortical vein (“cord sign”), an early but easily overlooked radiographic clue [5]. While previous reports have emphasized the diagnostic variability of isolated cortical vein thrombosis [3,4], bilateral thrombosis involving the superior anastomotic veins (veins of Trolard) has been described only rarely, with limited published cases available to characterize its clinical course or imaging evolution [6]. Because the veins of Trolard represent the dominant superficial anastomotic pathway between the superficial middle cerebral vein and superior sagittal sinus, bilateral involvement has the potential to compromise cortical venous drainage over a broad frontoparietal territory, increasing the risk of venous congestion, cortical dysfunction, and hemorrhagic complications despite initially subtle neurological findings. Advanced venous imaging modalities, particularly MR venography (MRV), are therefore essential for confirming the diagnosis when initial CT findings are subtle or nonspecific [7]. We present a rare case of bilateral vein of Trolard thrombosis (left greater than right) presenting predominantly with seizure despite a normal neurological examination at evaluation, highlighting that extensive bilateral cortical venous thrombosis may initially manifest with minimal persistent neurological deficits when recognized early.

## 2. Case Presentation

### 2.1. Patient Information

A 64-year-old right-handed man with no significant past medical history presented after being found unresponsive at home. According to family members, he entered the bathroom and was subsequently found on the floor with altered consciousness, raising concern for a possible seizure. Upon recovery, he reported slurred speech, imbalance, and mild right upper extremity weakness. He had no prior history of hypertension, diabetes mellitus, hyperlipidemia, atrial fibrillation, hypercoagulable disorders, malignancy, venous thromboembolism, or previous cerebrovascular events and was not taking anticoagulant or antiplatelet therapy. One week before presentation, he experienced fevers and intermittent chills attributed to a presumed sinus infection, for which antibiotics were prescribed but discontinued because of an allergic reaction. Although this recent illness raised the possibility of a transient prothrombotic or dehydration-related state, no definitive infectious or inflammatory etiology was established. There was no significant family history of thrombophilia, venous thromboembolism, stroke at a young age, or other similar thrombotic events.

#### Initial Clinical Course

The patient was last known well at approximately 7:20 PM and was subsequently found unresponsive. Bystander cardiopulmonary resuscitation was initiated because the patient was found unresponsive and pulselessness could not be confidently excluded. Upon emergency medical services arrival, spontaneous circulation was present, and he was transported to the emergency department. On arrival to the emergency department, vital signs were notable for hypertension (blood pressure 193/105 mmHg) and hyperglycemia (glucose 215 mg/dL). Laboratory evaluation revealed an elevated lactate level, supporting a possible preceding seizure. On neurological examination, the patient was awake, alert, and oriented, with fluent speech and resolution of the previously reported dysarthria. Cranial nerve examination, motor strength, sensation, coordination, and language were intact without focal deficits, consistent with an NIHSS score of 0. The neurological assessment consisted of a standard bedside examination during the acute evaluation; formal neuropsychological testing was not performed.

### 2.2. Diagnostic Workup and Treatment

At presentation, the differential diagnosis included acute ischemic stroke with possible large-vessel occlusion, primary seizure with postictal deficits (Todd paralysis), metabolic encephalopathy, and less likely cardiac syncope. The transient neurological symptoms and suspected seizure initially prompted an acute stroke evaluation with CT, CTA, and CT perfusion. The absence of arterial occlusion together with subtle bilateral cortical vein hyperdensity on noncontrast CT shifted suspicion toward cortical vein thrombosis, which was subsequently confirmed by MRI and MR venography.

**Figure 1:**
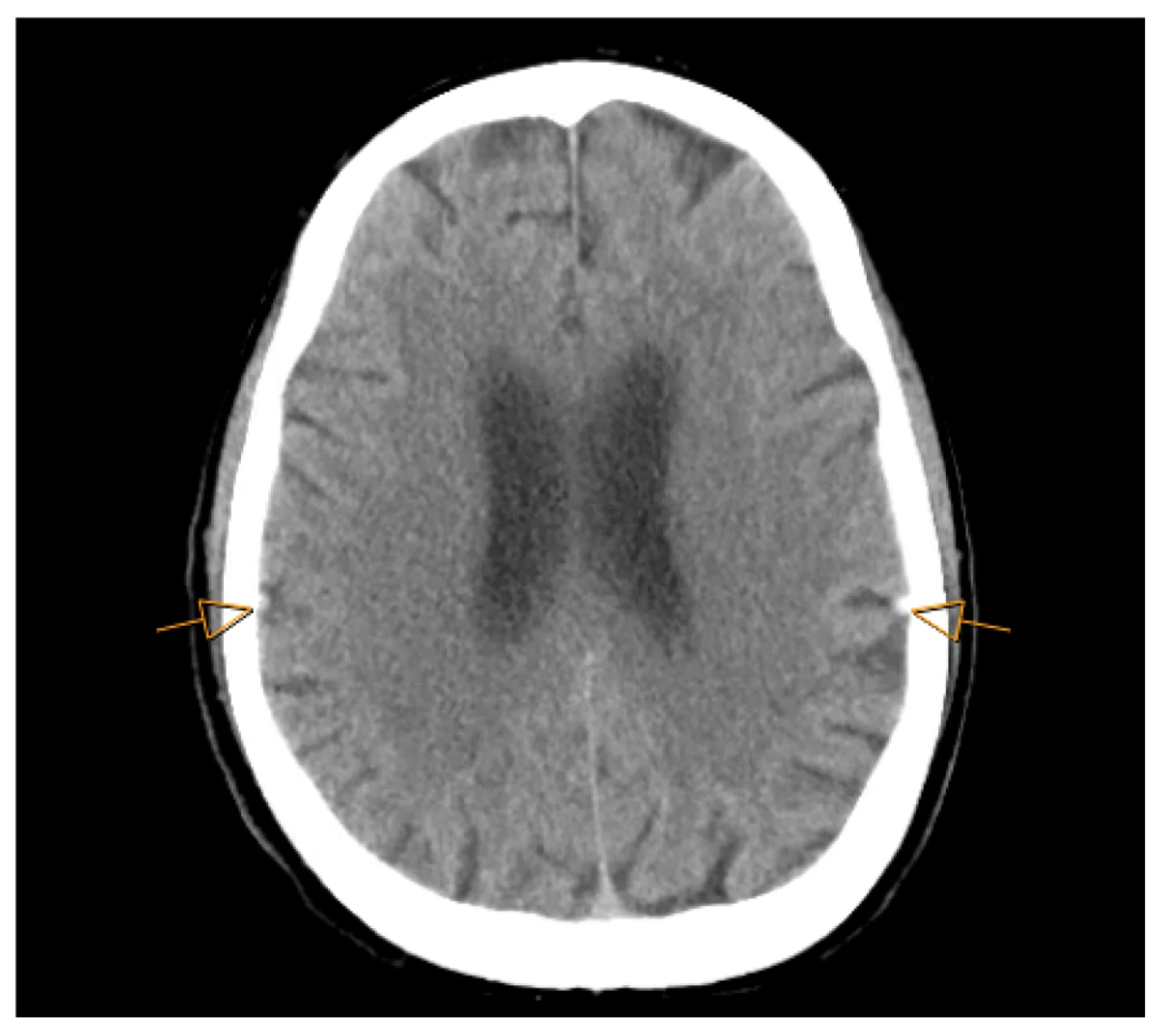
Axial noncontrast CT head demonstrating bilateral hyperdense cortical veins along the parietal convexities (arrows), more pronounced on the left.

Initial noncontrast computed tomography (CT) of the head, CT angiography (CTA) of the head and neck, and CT perfusion were performed approximately 2 h after the patient was last known well (9:23 PM). CTA demonstrated no evidence of large vessel occlusion or other acute arterial abnormality. However, the noncontrast CT demonstrated subtle hyperdense, mildly enlarged cortical veins along the bilateral frontoparietal convexities, more pronounced on the left, consistent with the “cord sign” and raising suspicion for cortical vein thrombosis (Figure 1). CTA further demonstrated relatively poor enhancement of these cortical veins compared with adjacent cortical veins, raising suspicion of cortical vein thrombosis.

The absence of large-vessel occlusion on CTA, together with persistent concern raised by the subtle bilateral cortical vein hyperdensity on noncontrast CT, prompted dedicated venous imaging with contrast-enhanced MRI and MR venography (MRV) to evaluate the cortical venous system.

MRI with MR venography was obtained approximately 45 min later (10:07 PM), confirming bilateral cortical vein thrombosis involving the superior anastomotic veins (veins of Trolard). Recognition of these subtle CT findings was critical, as the hyperdense cortical vein (“cord sign”) prompted timely dedicated venous imaging and definitive diagnosis [5].

CT perfusion performed during the initial stroke evaluation demonstrated no evidence of core infarction and therefore did not identify a significant perfusion deficit despite the presence of cortical vein thrombosis. Additional volumetric quantification of thrombus burden or venous congestion was not performed.

**Figure 2:**
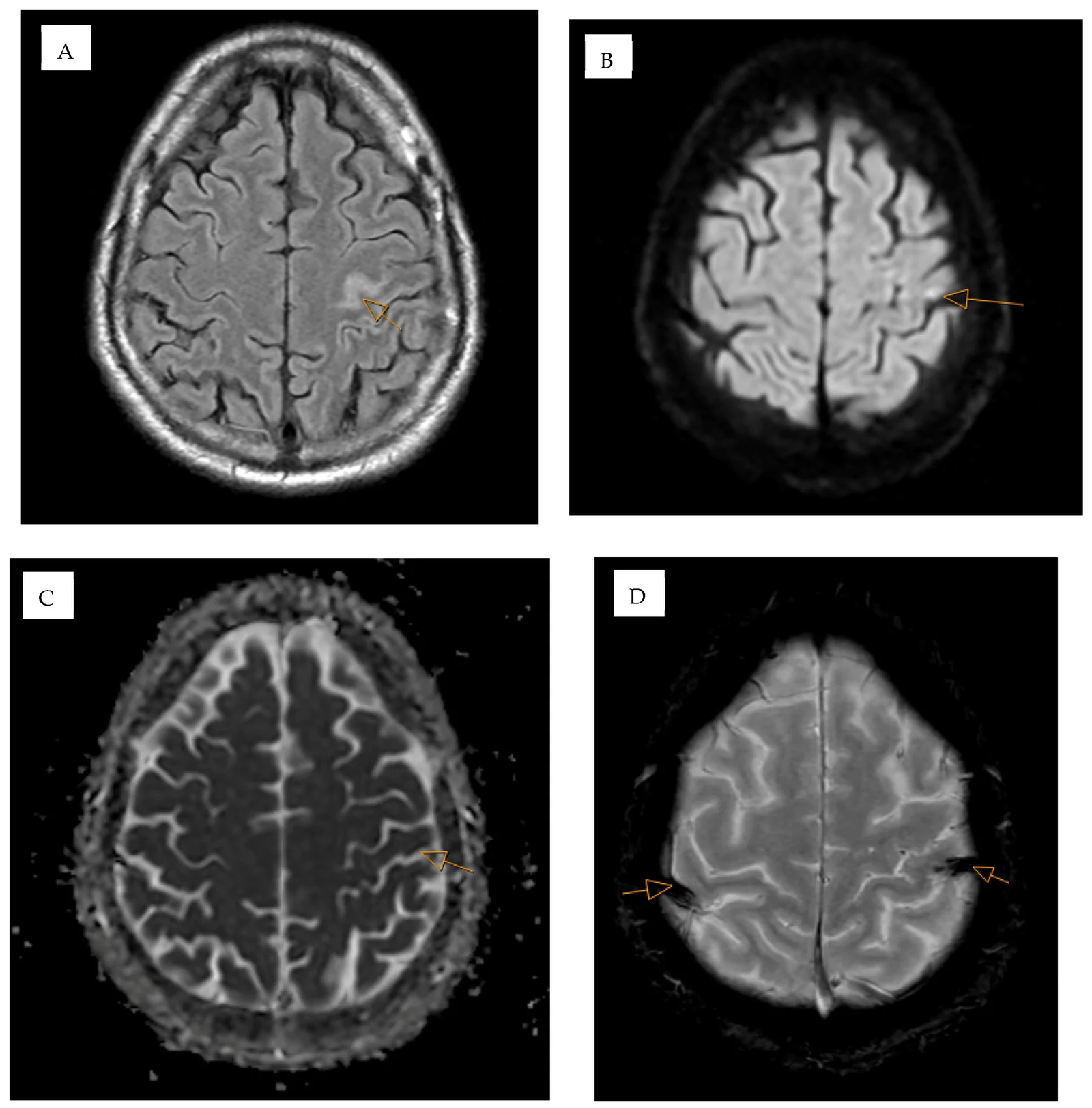
Multimodal MRI of the brain. (**A**) Axial fluid-attenuated inversion recovery (FLAIR) image demonstrating focal cortical hyperintensity in the left superior frontoparietal region (arrow). (**B**) Diffusion-weighted imaging (DWI) demonstrating corresponding cortical hyperintensity (arrow). (**C**) Apparent diffusion coefficient (ADC) map demonstrating corresponding cortical hypointensity (arrow). (**D**) Gradient-recalled echo (GRE) image demonstrating susceptibility within bilateral cortical veins (arrows), more pronounced on the left.

The patient presented with seizure and transient right-sided symptoms localizing to the left frontoparietal cortex, corresponding to thrombosis of the left vein of Trolard with imaging evidence of cortical venous congestion, early diffusion restriction, a small sulcal subarachnoid hemorrhage, and cortical FLAIR hyperintensity consistent with cortical irritation/postictal change (Figure 2A–D). Although the imaging demonstrated bilateral cortical vein thrombosis, no quantitative assessment of thrombus burden or venous congestion severity was performed. Diffusion-weighted imaging demonstrated corresponding hyperintensity with subtle hypointensity on the apparent diffusion coefficient (ADC) map, consistent with early diffusion restriction (Figure 2B,C). Gradient-recalled echo (GRE) imaging demonstrated susceptibility hypointensity along bilateral thrombosed cortical veins (Figure 2D).

**Figure 3:**
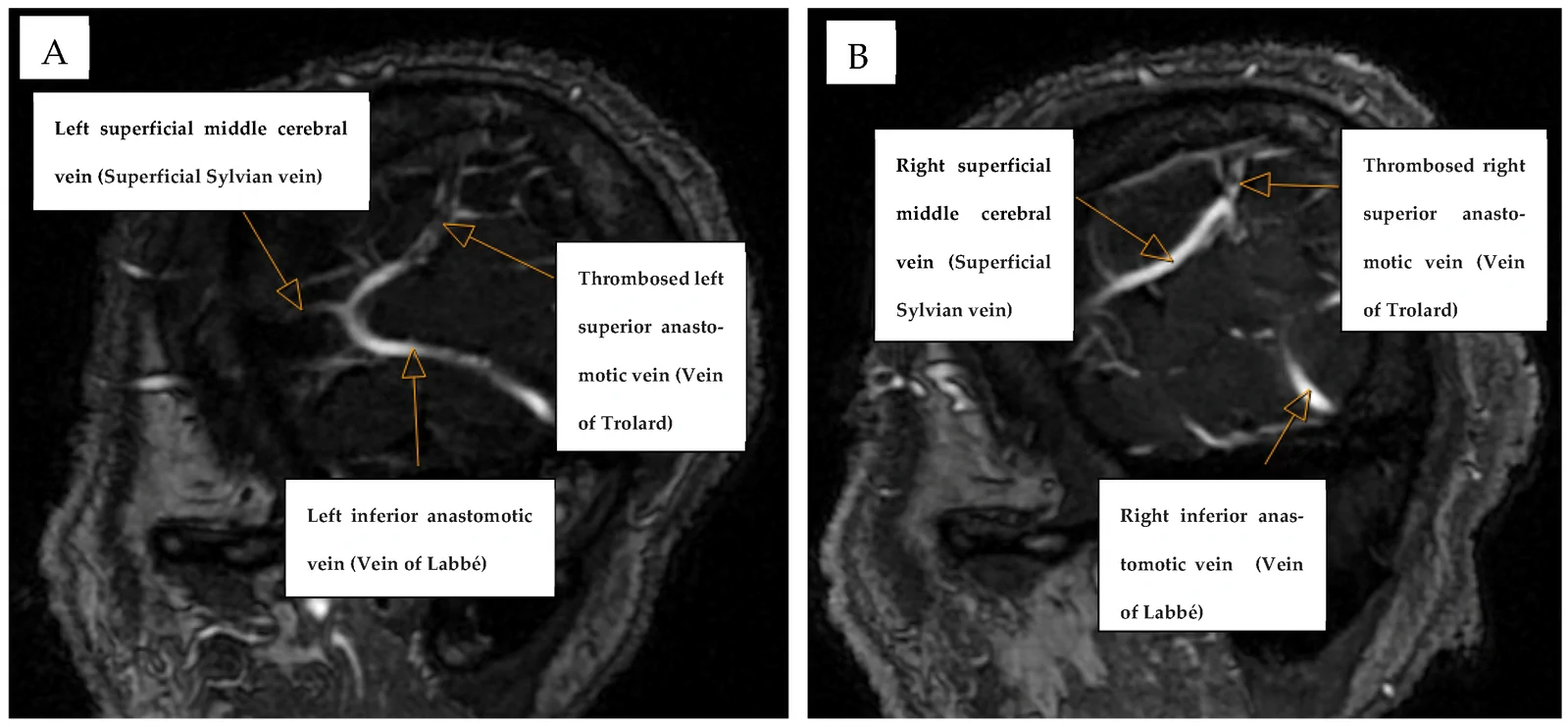
Contrast-enhanced sagittal three-dimensional MR venography (3D MRV). (**A**) Left parasagittal view and (**B**) right parasagittal view demonstrating the superficial middle cerebral vein (superficial Sylvian vein), inferior anastomotic vein (vein of Labbé), and superior anastomotic vein (vein of Trolard). Arrows indicate filling defects within the superior anastomotic veins.

Contrast-enhanced MR venography confirmed bilateral filling defects involving the superior anastomotic veins (veins of Trolard), establishing the diagnosis of bilateral cortical vein thrombosis (Figure 3). Bilateral involvement of the veins of Trolard is exceedingly rare and has been described only in limited reports, underscoring the uniqueness of this case [6].

A comprehensive hypercoagulable evaluation was performed, including testing for protein C and protein S activity, antiphospholipid antibodies, and prothrombin gene mutation, all of which were within normal limits. Evaluation for underlying thrombophilia is an important component of CVT workup, as inherited or acquired hypercoagulable states may contribute to pathogenesis [8]. Although no definitive infectious etiology was established, the recent presumed sinus infection may have represented a transient prothrombotic trigger, consistent with previous reports describing infection-associated CVT [9]. In the absence of inherited thrombophilia or other persistent provoking factors, the overall presentation was most consistent with a transient provoked cortical vein thrombosis.

The patient was initiated on therapeutic anticoagulation with enoxaparin at a dose of 1 mg/kg subcutaneously every 12 h for five days, followed by transition to apixaban 5 mg orally twice daily with a planned duration of six months. Anticoagulation was pursued despite the presence of a small sulcal subarachnoid hemorrhage because isolated convexity or sulcal subarachnoid hemorrhage in cerebral venous thrombosis is typically secondary to venous hypertension rather than aneurysmal rupture and is not considered a contraindication to therapeutic anticoagulation according to current European Stroke Organization recommendations [1]. Following initial treatment with therapeutic enoxaparin, apixaban was selected for long-term anticoagulation because of its fixed dosing, lack of routine laboratory monitoring, favorable safety profile, and expected improvement in treatment adherence compared with warfarin. Although the randomized RE-SPECT CVT trial evaluated dabigatran rather than apixaban, it demonstrated that direct oral anticoagulants are a reasonable alternative to vitamin K antagonists in selected patients with cerebral venous thrombosis [10]. In addition, observational studies, including the ACTION-CVT study, have demonstrated comparable efficacy with lower rates of major hemorrhage among patients treated with direct oral anticoagulants, supporting the use of apixaban in contemporary clinical practice [11]. For seizure management, he received a levetiracetam loading dose of 2000 mg intravenously for an acute symptomatic seizure, followed by maintenance dosing of 750 mg orally twice daily, with plans for reassessment of continued need at follow-up rather than indefinite prophylaxis. Anticoagulation remains the cornerstone of treatment for cortical vein thrombosis and is associated with favorable outcomes when initiated promptly, even in the presence of hemorrhagic findings [1,4].

## 3. Follow-Up

The patient remained neurologically stable throughout hospitalization without recurrent seizures or development of focal neurological deficits. Repeat CT imaging demonstrated stability of the small sulcal subarachnoid hemorrhage without progression. He was discharged on apixaban and levetiracetam with a modified Rankin Scale (mRS) score of 0.

At the 3-month follow-up, the patient remained neurologically intact (mRS 0) without recurrent seizures or new neurological symptoms. He reported no cognitive complaints, and bedside clinical evaluation demonstrated intact language, attention, memory, and executive functioning; formal neuropsychological testing was not performed. Follow-up MRI demonstrated interval improvement of the previously identified left central sulcal FLAIR hyperintensity, consistent with resolution of the acute cortical venous insult. Follow-up MR venography demonstrated significant interval improvement in the bilateral vein of Trolard thromboses, with only short-segment residual nonocclusive filling defects (approximately 3 cm on the right and 2 cm on the left), indicating partial venous recanalization. These radiographic findings paralleled the patient’s favorable clinical recovery and supported continued anticoagulation. Given the potential for delayed complications following cortical vein thrombosis, including the rare development of dural arteriovenous fistula, continued clinical and radiographic surveillance was recommended.

## 4. Discussion

This case illustrates that the bilateral vein of Trolard thrombosis may present predominantly with seizure despite a normal neurological examination. Unlike the more typical presentation of cortical vein thrombosis with headache, focal deficits, or venous infarction, isolated seizure may be the sole manifestation, increasing the risk of delayed diagnosis [3,4]. Delayed recognition may allow progression of venous congestion and, in rare cases, require surgical intervention, as illustrated by Bai et al., whose patient underwent thrombectomy after isolated cortical vein thrombosis was initially mistaken for an intracranial neoplasm [12].

Hyperdense cortical veins on noncontrast CT, commonly referred to as the “cord sign,” represent an early but easily overlooked radiographic clue that should prompt dedicated venous imaging when arterial studies are unrevealing [5]. In this patient, the subtle bilateral cortical vein hyperdensities were subsequently reviewed with a board-certified neuroradiologist after discussion on the clinical presentation, and a consensus was reached in that the findings were consistent with the cord sign. This interpretation was subsequently corroborated by contrast-enhanced MRI and MR venography demonstrating bilateral thrombosis of the veins of Trolard. Although quantitative assessment of thrombus burden or venous congestion was not performed, the combination of noncontrast CT, MRI, GRE, and MR venography established the diagnosis and guided management. Bilateral involvement of the superior anastomotic veins likely reflects more extensive impairment of superficial cortical venous drainage and may increase the risk of venous congestion and cortical dysfunction despite minimal neurological deficits [6].

The etiology of cortical vein thrombosis is frequently multifactorial and warrants systematic evaluation for inherited and acquired prothrombotic disorders. Common causes include thrombophilias, malignancy, inflammatory disorders, pregnancy, oral contraceptive use, trauma, and local or systemic infections [1,2]. In our patient, an extensive hypercoagulable evaluation, including protein C and protein S activity, antiphospholipid antibodies, and prothrombin gene mutation testing, was unrevealing, and there was no history of malignancy or other established thrombotic risk factors. The preceding presumed sinus infection was considered a possible transient trigger based on temporal proximity, although this association could not be corroborated by inflammatory biomarkers, as C-reactive protein, erythrocyte sedimentation rate, and D-dimer were not obtained during the acute evaluation. Nevertheless, infection-associated endothelial activation and transient hypercoagulability have been described as potential mechanisms contributing to cerebral venous thrombosis [9]. This case further illustrates that cortical vein thrombosis may occur even when routine thrombophilia testing is negative, reinforcing the importance of integrating clinical history with dedicated venous imaging when clinical suspicion remains high.

Anticoagulation remains the cornerstone of treatment for cortical vein thrombosis and is recommended even in the presence of small venous hemorrhagic lesions because these typically reflect venous hypertension rather than a contraindication to therapy [1,4]. Historically, vitamin K antagonists were considered the standard long-term anticoagulant following initial heparin therapy. However, emerging evidence supports direct oral anticoagulants as reasonable alternatives. The randomized RE-SPECT CVT trial demonstrated comparable efficacy and safety of dabigatran and warfarin, while the multicenter ACTION-CVT study provided additional real-world evidence supporting DOACs, including apixaban, as alternatives to warfarin [10,11]. More recently, a multicenter study specifically comparing apixaban with vitamin K antagonists found no significant differences in recanalization, favorable functional outcomes, intracranial hemorrhage, mortality, or recurrent cerebral venous thrombosis [13]. Although randomized trial evidence specifically evaluating apixaban remains lacking, its selection in this patient was supported by the available observational evidence, absence of contraindications, favorable safety profile, and practical advantage of avoiding routine INR monitoring.

Compared with the limited published reports of the bilateral vein of Trolard thrombosis, our patient demonstrated a similarly favorable outcome despite an initially subtle clinical presentation. Mishra et al. reported that bilateral thrombosis of the veins of Trolard is exceptionally uncommon and may present with seizures and relatively limited focal neurological deficits despite bilateral cortical venous involvement [6]. Similar to the present case, the patient reported by Mishra et al. recovered favorably following anticoagulation despite bilateral cortical venous involvement [6]. In contrast to Bai et al., whose patient underwent delayed diagnosis and ultimately required surgical thrombectomy after cortical vein thrombosis was initially mistaken for an intracranial neoplasm [12], recognition of the subtle noncontrast CT cord sign in our patient prompted early MRI and MR venography, timely anticoagulation, and complete neurological recovery. Follow-up MRI demonstrated interval resolution of the previously identified cortical FLAIR abnormality, while follow-up MR venography demonstrated significant regression of the bilateral thrombi with residual short-segment nonocclusive filling defects, consistent with partial bilateral recanalization. Long-term clinical and radiographic follow-up remains important after cortical vein thrombosis to assess thrombus resolution, guide anticoagulation duration, and identify delayed complications such as recurrent thrombosis, post-thrombotic epilepsy, or the uncommon development of dural arteriovenous fistula [14].

To our knowledge, this is among the few reported cases of bilateral vein of Trolard thrombosis with serial MRI and MR venography demonstrating interval thrombus regression and partial bilateral recanalization, providing insight into the radiographic evolution of this exceptionally rare entity.

## 5. Conclusions

This case represents one of the few reported cases of bilateral vein of Trolard thrombosis and expands the recognized clinical spectrum by demonstrating that extensive bilateral cortical venous thrombosis may present with seizure and minimal neurological deficits despite radiographic evidence of venous congestion, early diffusion restriction, and a small sulcal subarachnoid hemorrhage. Recognition of subtle imaging findings, particularly the noncontrast CT “cord sign,” should prompt dedicated venous imaging with MRI and MR venography when cortical vein thrombosis is suspected. Early diagnosis and prompt anticoagulation, even in the presence of a small associated venous hemorrhage, may result in excellent neurological recovery and interval thrombus regression with partial venous recanalization.

## Data Availability

Data supporting the findings of this study are contained within the article. Further data are not publicly available due to patient privacy and ethical restrictions.
